# Swarms of the malaria vector *Anopheles funestus* in Tanzania

**DOI:** 10.1186/s12936-019-2660-y

**Published:** 2019-01-29

**Authors:** Emmanuel W. Kaindoa, Halfan S. Ngowo, Alex J. Limwagu, Magellan Tchouakui, Emmanuel Hape, Said Abbasi, Japhet Kihonda, Arnold S. Mmbando, Rukiyah M. Njalambaha, Gustav Mkandawile, Hamis Bwanary, Maureen Coetzee, Fredros O. Okumu

**Affiliations:** 1 Environmental Health and Ecological Science Department, Ifakara Health Institute, P. O. Box 53, Ifakara, Tanzania; 2 School of Public Health, Faculty of Health Sciences, University of the Witwatersrand, Johannesburg, South Africa; 3 Centre for Research in Infectious Diseases (CRID), P.O. BOX 13591, Yaoundé, Cameroon; 4 School of Pathology, Faculty of Health Sciences, Wits Research Institute for Malaria and Wits/MRC Collaborating Centre for Multidisciplinary Research on Malaria, University of the Witwatersrand, Johannesburg, South Africa; 5 Centre for Emerging Zoonotic & Parasitic Diseases, National Institute for Communicable Diseases, Johannesburg, South Africa; 6 Institute of Biodiversity, Animal Health and Comparative Medicine, University of Glasgow, Glasgow, G12 8QQ UK

## Abstract

**Background:**

*Anopheles funestus* mosquitoes currently contribute more than 85% of ongoing malaria transmission events in south-eastern Tanzania, even though they occur in lower densities than other vectors, such as *Anopheles arabiensis*. Unfortunately, the species ecology is minimally understood, partly because of difficulties in laboratory colonization. This study describes the first observations of *An. funestus* swarms in Tanzania, possibly heralding new opportunities for control.

**Method:**

Using systematic searches by community-based volunteers and expert entomologists, *An. funestus* swarms were identified in two villages in Ulanga and Kilombero districts in south-eastern Tanzania, starting June 2018. Swarms were characterized by size, height, start- and end-times, presence of copulation and associated environmental features. Samples of male mosquitoes from the swarms were examined for sexual maturity by observing genitalia rotation, species identity using polymerase chain reaction and wing sizes.

**Results:**

581 *An. funestus* (98.1% males (n = 570) and 1.9% (n = 11) females) and 9 *Anopheles gambiae* sensu lato (s.l.) males were sampled using sweep nets from the 81 confirmed swarms in two villages (Ikwambi in Kilombero district and Tulizamoyo in Ulanga district). Six copulation events were observed in the swarms. Mean density (95% CL) of *An. funestus* caught/swarm/village/evening was 6.6 (5.9–7.2) in Tulizamoyo and 10.8 (5.8–15.8) in Ikwambi. 87.7% (n = 71) of the swarms were found in Tulizamoyo, while 12.3% (n = 10) were in Ikwambi. Mean height of swarms was 1.7 m (0.9–2.5 m), while mean duration was 12.9 (7.9–17.9) minutes. The PCR analysis confirmed that 100% of all *An. funestus* s.l. samples processed were *An. funestus* sensu stricto. Mean wing length of *An. funestus* males was 2.47 mm (2.0–2.8 mm), but there was no difference between swarming males and indoor-resting males. Most swarms (95.0%) occurred above bare ground, sometime on front lawns near human dwellings, and repeatedly in the same locations.

**Conclusion:**

This study has demonstrated occurrence of *An. funestus* swarms for the first time in Tanzania. Further investigations could identify new opportunities for improved control of this dominant malaria vector, possibly by targeting the swarms.

## Background

Global statistics indicate that malaria morbidity and mortality have declined mostly as a result of scaling up vector control interventions [1, 2], but that the gains are stagnating in some countries [3]. This decline has also been observed in Tanzania with more than 50% reduction of malaria burden recorded since the year 2000 [4]. The 2016–2017 Tanzania malaria indicator survey demonstrated reduction of prevalence in children under five, from 18.0% in 2008 to 7.3% in 2017 [5–7] in the mainland. These successes may be attributed to scaling up of vector control tools, such as long-lasting insecticide-treated bed nets [8] and indoor residual sprays [9], as well as socio-economic developments and urbanization [10] and treatment with artemisinin-based combination therapy (ACT) [11].

In a recent study, it was demonstrated that just one malaria vector species, *Anopheles funestus,* contributes to more than 85% of malaria transmission in south-eastern Tanzania, despite occurring at far lower densities than the other major vector, *An. arabiensis* [12]. Unfortunately, the *An. funestus* populations are resistant to pyrethroid insecticides commonly-used on bed nets [12, 13], survive longer than *An. arabiensis,* and feed almost exclusively on humans [12, 14]. Other studies in different African countries have also documented *An. funestus* resistance to pyrethroids: Malawi [15, 16], Mozambique [17, 18], South Africa [19], Zambia [20, 21], Zimbabwe [21], Cameroon [22, 23] and Senegal [24], a situation that compromises effectiveness of current vector control options [25], and perpetuates residual transmission even in communities where bed net coverage is more than 90% [26]. Given its dominance in Tanzania, it has been suggested that interventions that effectively target *An. funestus* could have a high impact on residual transmission here [12]. One potentially effective approach involves suppressing mosquito populations by identifying and directly targeting *Anopheles* swarms with highly effective insecticides [27], before the mosquitoes enter houses.

Swarming behaviours have previously been intensively studied, mostly in Western and Central Africa [28–30]. However in East and southern Africa, there have only been a few studies in Zambia and Mozambique [20, 31] in addition to an old set of observations in northern Tanzania in the 1980s [32]. *Anopheles* swarms are naturally difficult to find since they occur at dusk when visibility is poor and last only for a few minutes. In fact, the rarity of *Anopheles* swarms in East Africa has led some to hypothesise that they mate primarily indoors, such as previously observed in experiments by Dao et al. [33]. Nevertheless, a recent study by the Ifakara Health Institute, which relied primarily on community volunteers rather than experts, demonstrated natural occurrence of swarms of *An. arabiensis* in villages across south-eastern Tanzania [34], more than three decades after the last records of this species swarming anywhere in the region [32]. In addition to providing detailed characterization of over 200 *An. arabiensis* swarms, the study also demonstrated that trained volunteers were able to identify and locate mosquito swarms in their villages [34]. Follow-up studies have since demonstrated potential for localized control by targeting the swarms using aerosol spraying (Kaindoa et al. unpublished data).

An interesting revelation in that last study on *An. arabiensis* swarms was a single incidence where 13 *An. funestus* males were caught in a sweep net [34], providing earliest indications that this species too formed swarms in the valley, but that these swarms were certainly more elusive than those of *An. arabiensis*. The aim of this current study was, therefore, to identify and characterize swarms of *An. funestu*s, which is now the dominant malaria vector species in rural south-eastern Tanzania.

## Methods

### Study area

**Fig. 1:**
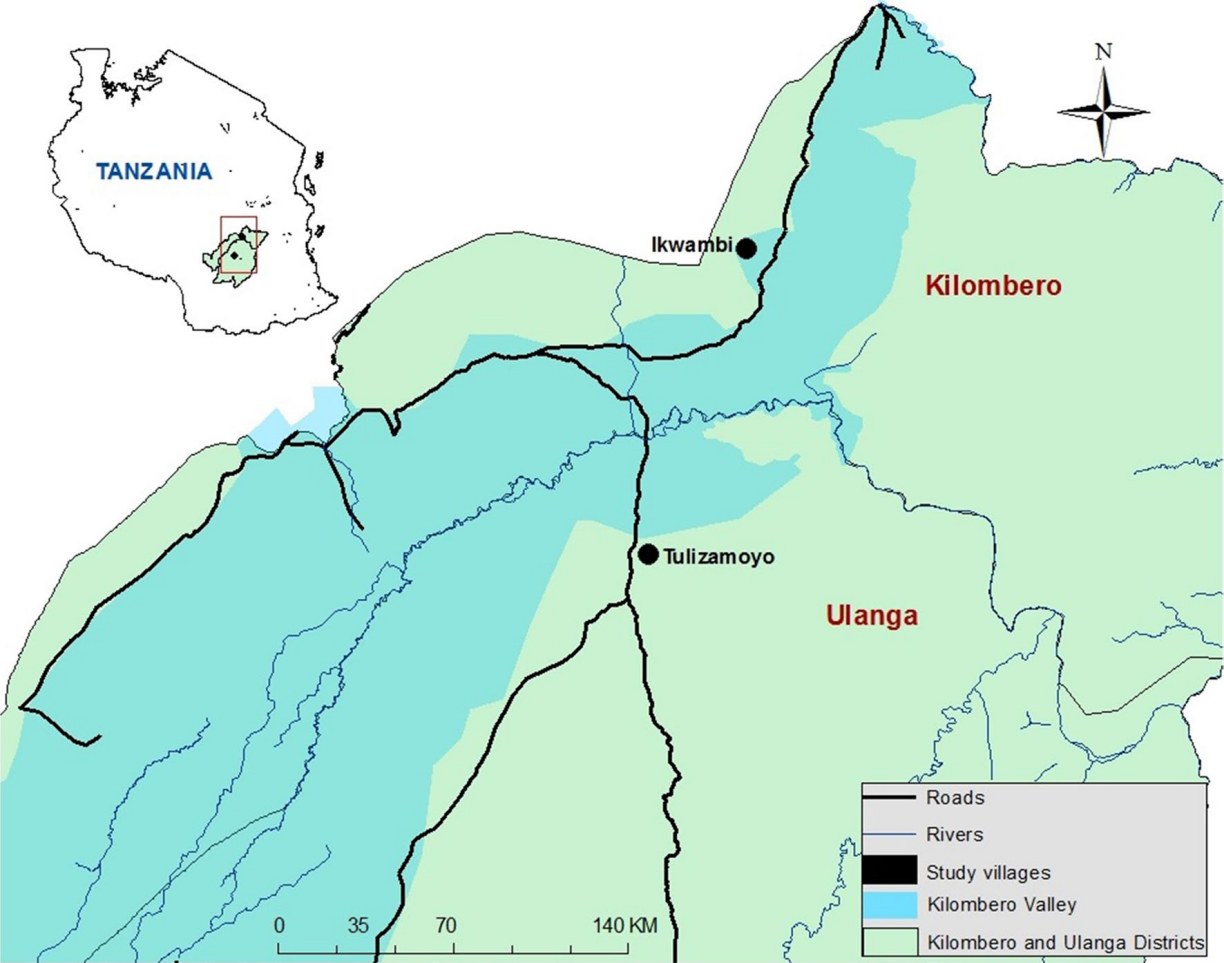
Map of the study area, showing villages in south-eastern Tanzania, where swarm surveys were conducted

The study was done in the two districts of Ulanga and Kilombero in south-eastern Tanzania. In Ulanga, collections were done in Tulizamoyo village (8.354497°S, 36.705468°E), while in Kilombero, the collections were in Ikwambi village (7.98244°S, 36.82167°E), both of them in the Kilombero river valley (Fig. 1). The villages were selected because previous adult mosquito surveys had yielded high densities of *An. funestus*. The area is characterized by perennial meso-endemic malaria transmission, with mosquito densities peaking between February and May [35]. Malaria vectors comprise primarily *An. arabiensis* and *An. funestus*, the former being more abundant but the latter more dominant in transmission.

### Observation and characterization of swarms

Following the successful training of village volunteers during the *An. arabiensis* swarm identifications in 2016 and 2017, a refresher training course was provided in early 2018 to the same volunteers, using the same procedures for swarm searching [34]. In this approach, the study villages were divided into sub-sections where volunteers had to first identify potential swarm markers, and then search for swarms later in the evening. Whenever a swarm was seen, volunteers reported to the supervisors and after physical expert verification, swarm sampling and characterization was performed the following day. Sampling was done 10–15 min after the start of swarming using standardized sweep nets (190 cm diameter attached to a 2 m long stick) as describe by Kaindoa et al. [34] and the sweeping was done only once per swarm. Similar sampling procedure was done across all the swarming events to allow comparison. Using a standard observation sheet, all the identified swarms were characterized as shown in Table 1.

**Table 1:** Descriptive variables assessed during field surveys of *Anopheles funestus* swarms

	Variables	Methods used and indicators measured
1	Swarm size and copulation events	Visual estimates of swarm sizes: approximate number of mosquitoes in swarm, as estimated visually to the nearest 5 mosquitoes (observations were made between 10 and 15 min after start of the swarm)
		Sweep net estimates of swarm sizes: approximate number of mosquitoes in the swarm, as estimated using standardized sweep nets, collected once by experienced collectors. Swarm size was a measure of density of mosquitoes per collection by sweep net per instance
		Copulation: number of copulation events observed in the swarm after 10 min of observation
2	Location, time and height of swarms	Geo-location of the swarm measured using handheld GPS receivers
		Unique ID of compound owner (each swarm was uniquely identified on this parameter)
		Time of day when the swarm begins appearing, recorded to the nearest minute
		Time of day when the swarms completely disperse, also measured to the nearest minute
		Height measured as distance between the base of the swarm and the ground level in meters
3	Molecular identification and characteristics of sampled mosquitoes	Morphological and molecular identification of the species of *Anopheles* mosquitoes collected in the swarm
		Proportion of males caught that have evidence of being capable of mating (determined by observing the rotation of male genitalia)
		Measurements of wing size (mm)
4	Important landmarks and potential swarm markers	A record of important landmarks, at or near which swarms occur such as vegetation, house, mosques, markets, schools, water pumps, houses, cowshed, banana tree and cemeteries

### Morphological and molecular identification of *Anopheles* mosquitoes collected in swarms

All collected mosquitoes were aspirated from the sweep nets, placed in paper cups and maintained on 10% glucose solution. The following morning, the mosquitoes were killed in a closed container by freezing, then identified morphologically by taxa and sex following keys by Gillies and Coetzee [36]. All samples from each collection were further identified by multiplex polymerase chain reaction (PCR) to sibling species [37].

### Assessing sexual maturity and wing sizes of sampled mosquitoes

All collected *An. funestus* males were assessed for maturity through observations of genitalia rotation as previously described by Dahan et al. [38]. Wing lengths of male mosquitoes sampled from swarms or resting indoor surfaces, were measured under a dissecting microscope. An additional 70 mosquitoes were collected from indoor surfaces of local houses using mouth aspirator and used for comparative assessment of wing size. These samples were collected in the same period as the samples from swarms. One wing of each specimen was cut and placed onto a slide, then its length measured from the alula notch to wing tip following procedures by Lyimo et al. [39] and Charlwood et al. [31].

### Data analysis

Data analysis was done by using R software version 3.3.2 [40]. Swarm sizes were calculated as median number of mosquitoes sampled in each swarm (using a sweep net when the swarming was at its peak), and compared between the two study villages. The swarm size was, therefore, a measure of mosquito densities in each sweep net collection. Swarm duration was obtained based on the difference between start and end times of each swarm in minutes. Mean height of swarms above ground, and mean duration of swarms were also calculated. Wing sizes of swarming and resting *An. funestus* males were compared. Predicted means from generalized linear model (glm) were used to produce different figures showing variations in catches between study sites. In this model, the number of mosquitoes caught in the swarms was modelled as count data following a Poisson distribution while study sites were considered as a predictor variable. Student’s T-test was used to compare the mean wing length of swarming and resting *An. funestus*. Geo-locations of the swarms were visualized in ArcGIS 10.4 (ESRI, USA). Swarm sizes estimated visually were compared to those estimated by using sweep nets and their correlation coefficients computed.

## Results

### Mean density of *Anopheles funestus* and copulation events in swarms

**Fig. 2:**
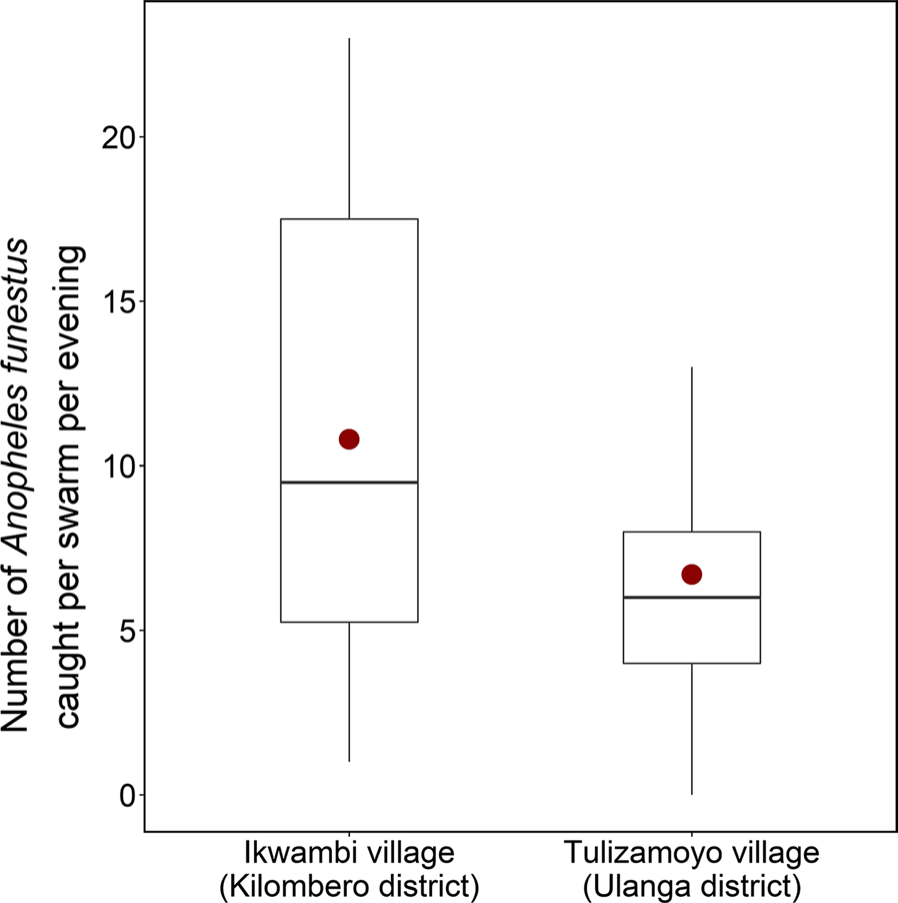
Median number of *An. funestus* caught per swarm per evening in the two study sites. The fitted model was generalized linear model (glm) fitted to obtained the predicted means used to plots the graphs showing the means catch between study villages

A total of 570 *An. funestus* males and 11 females were sampled using sweep nets, from 81 swarms observed in the two villages. A total of 6 copulation events were observed in the swarms, following the standardized observation period of 10 min per swarm (Table 1). Mean density of *An. funestus* caught per swarm per village per evening was 6.6 (5.7–7.5) in Tulizamoyo and 10.8 (5.8–15.8) in Ikwambi (Fig. 2). Overall, 87.7% [n = 71] of the swarms were found in Tulizamoyo, while only 12.3% [n = 10] were in Ikwambi. Four instances of mixed swarms were identified in Tulizamoyo village which contained 2, 3, 1, and 3 *Anopheles gambiae* sensu lato male mosquitoes found together in the same sweep nets with 2, 2, 3 and 7 *An. funestus* males, respectively, suggesting either mixed swarms or swarms of different species appearing in close proximity.

### Correlations between visual and sweep net estimates of swarm sizes

**Fig. 3:**
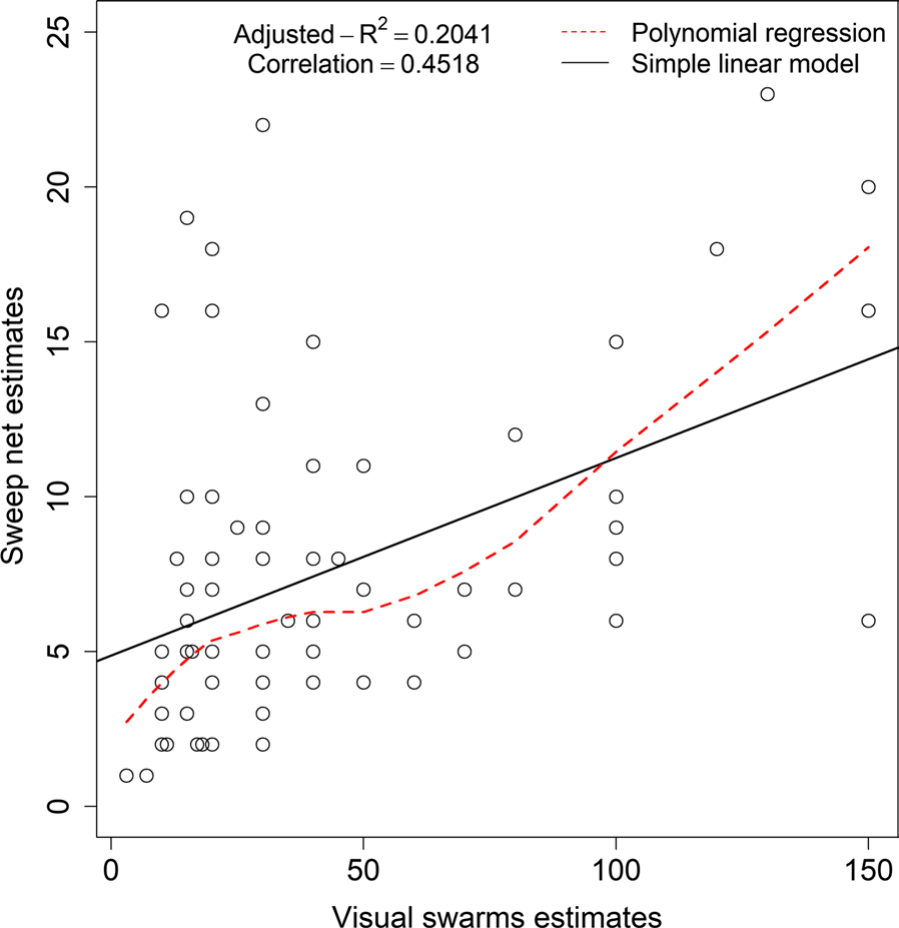
Correlations between visual estimates of the swarm sizes and the swarm densities per sweep net collection per evening

*Anopheles funestus* swarm size was estimated by visual assessments of approximate numbers of mosquitoes forming the swarm prior to sampling with sweep nets (as described in Table 1) to estimate mosquito densities per sweep. Regression models revealed significant but weak correlation between visual estimates and sweep net estimates (Linear regression analysis: R = 0.4518, P-value < 0.001; Polynomial regression: Adjusted R^2^ = 0.2041, P-value < 0.001) (Fig. 3).

### Location, time and height at which the swarms occurred

The identified swarms were all characterized in the months of June and July 2018 and are shown in a georeferenced map in Fig. 5. Most swarms started at approximately 6:40 pm. Mean (± SD) duration of the swarm was 12 (± 5) minutes. The mean height of swarms above ground was 1.7 (± 0.8) meters. It was observed that most *An. funestus* swarms occurred close to human houses.

### Environmental features associated with swarm occurrence

**Fig. 4:**
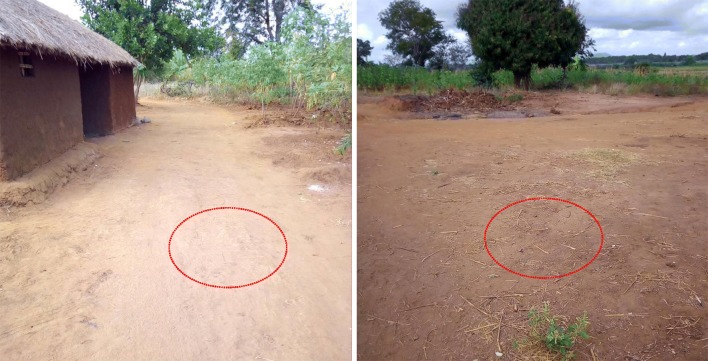
Pictorial illustration of commonest locations where swarms of *Anopheles funestus* mosquitoes were observed

**Fig. 5:**
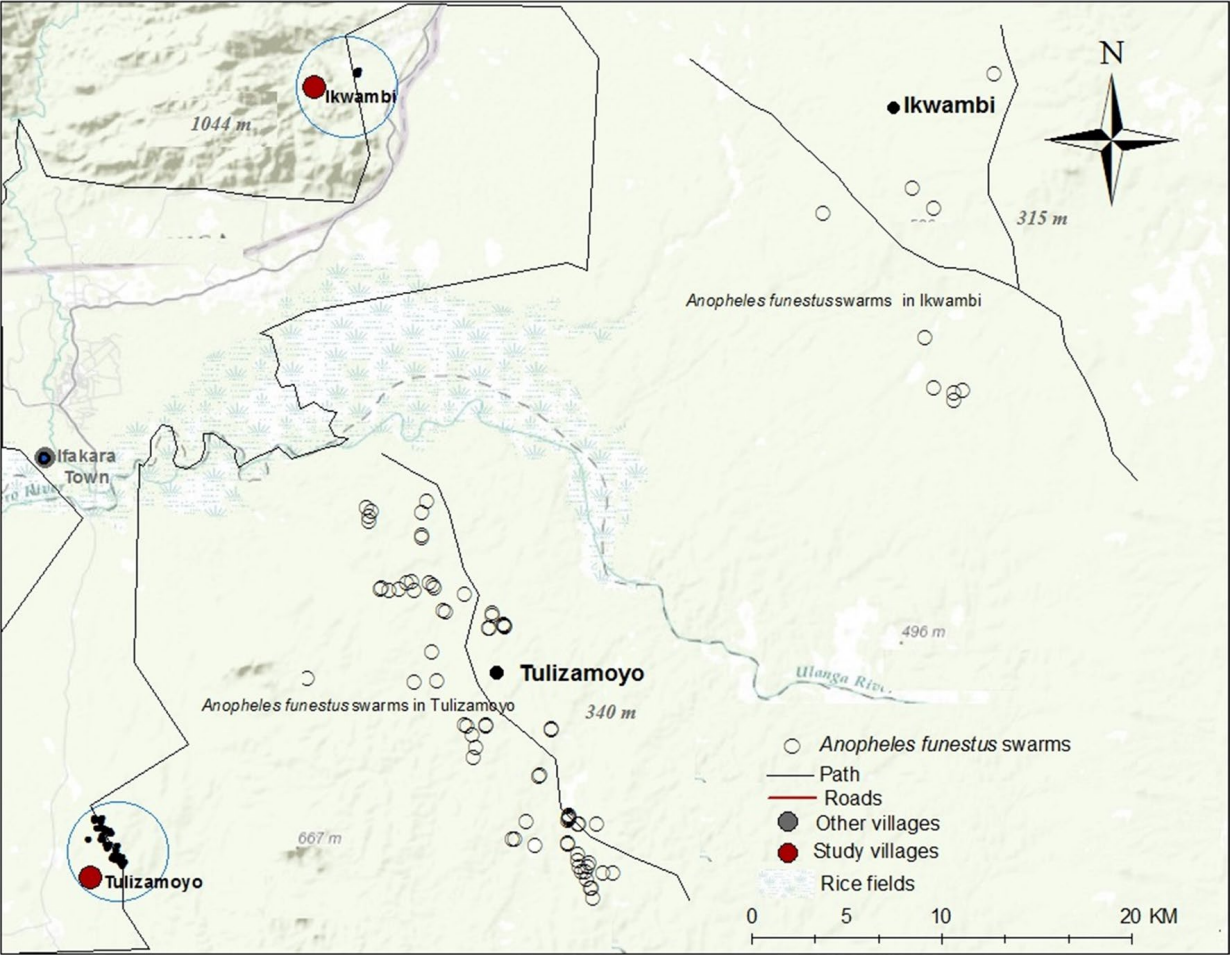
Distribution of *Anopheles funestus* swarms observed in study sites

There were no obvious physical features being used as markers as previously described for *An. arabiensis*. However, assessment of all the swarm locations resulted in four different categories of places where swarming by *An. funestus* occurred. The most common feature was bare ground near the houses (Fig. 4), usually dusty but no obvious textural discontinuation and sometimes in the front lawns of human dwellings (91.4% (n = 74) of the swarms occurred in such sites) (Fig. 5). Two swarms were observed on small cleared farms (2.5% of the swarms; (n = 2), one swarm under of a teak tree, and one over a demolished house (Table 2).

**Table 2:** Environmental features of locations where *Anopheles funestus* swarms were observed in the study villages

	Environmental features	Total marker (%)	No. mosquitoes caught
1	Bare ground near dwellings	77 (95.0)	535
2	Demolished house	1 (1.2)	18
3	Cleared farms	2 (2.5)	10
4	Teak tree	1 (1.3)	18
	Total	81 (100)	581

### Molecular identification, wing lengths and sexual maturity of sampled *An. funestus*

**Fig. 6:**
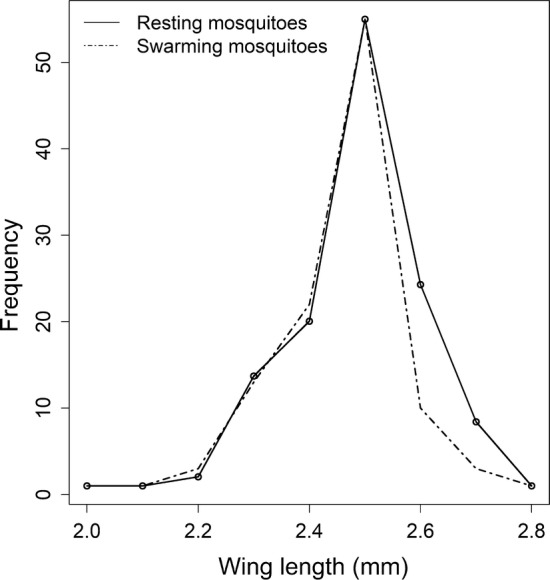
Comparison of wing sizes between swarming and resting *Anopheles funestus* male mosquitoes

A total of 413 *An. funestus* males were analysed for species identification in the laboratory, 93.9% (n = 388) of which yielded successful DNA amplifications. All amplified samples were confirmed as *An. funestus* s.s. A total of 109 and 70 swarming and resting *An. funestus* males, respectively, were examined for size, using wing length as proxy. The wing length of *An. funestus* males ranged between 2.00 and 2.80 mm with a mean of 2.47 mm (Minimum = 2.00 and Maximum = 2.80) as shown in Fig. 6. There was a very small insignificant difference in wing size between the swarming and resting male *An. funestus* (t = 2.301, df = 214.38, P = 0.022). Nearly all (97.3%) of the male *An. funestus* had rotated genitalia, suggesting sexual maturity.

## Discussion

Improved understanding of malaria vector ecology and behaviour is crucial for achieving the ambition of malaria elimination through vector control tools [41]. The swarming and mating behaviours in particular are components of mosquito behaviour that has been neglected for a long time [30, 31, 34]. Given that *An. funestus* contributes to more than 80% of the ongoing malaria transmission in the area, it is important to identify and characterize swarms of *An. funestus,* so as to explore complimentary tools, possibly targeting swarming behaviour.

Furthermore, it is important to understand mating behaviour for implementation of certain control methods, e.g. use of sterile insect techniques and release of genetically modified males. This is the first verified report of *An. funestus* swarms in Tanzania, though the earlier study confirmed natural occurrence of *An. arabiensis* swarms in the same area [34]. Together, the two reports confirm widespread occurrence of swarms by the main malaria vectors, even though they are generally elusive and require dedicated teams with local knowledge to map.

An attempt to characterize the swarm sites did not yield any obviously distinctive physical markers of the swarm stations. Instead, most of the swarms were observed to occur above bare ground, sometime on the front lawns of human houses (Fig. 4). This is very similar to observations by Charlwood et al. who suggested that *An. funestus* mosquitoes in Mozambique formed swarms close to the houses used for resting and that the swarming sites could be used as indicators of houses to be targeted for vector control interventions [31].

Proximity to houses is therefore an essential condition for *An. funestus* mating swarms, and the possibility that some degree of mating happens indoors cannot be excluded. Relative to previous observations in the same area [34], this current study has demonstrated that *An. funestus* swarms differ from those of *An. arabiensis* in terms of height, swarm size and location. For example, while *An. arabiensis* males were observed swarming at mean heights of 2.5 m, swarms of *An. funestus* occurred at much lower heights averaging just 1.7 m above ground, with several swarms lower than 1 m. In terms of location and markers, *An. funestus* preferred to swarm very close to human houses, unlike *An. arabiensis* swarms, which were mostly at the edge of the villages [34], possibly due to the greater anthropophily of the former than the latter species. The *An. funestus* swarms were small and generally consisted of less than 15 mosquitoes, as collected by sweep nets, while *An. arabiensis* swarm sizes ranged from approximately 10 to 60 mosquitoes [34]. It is possible that these differences in vector densities may be associated with season (this *An. funestus* study was conducted between June and July 2018 while the *An. arabiensis* study was conducted between August 2016 and June 2017). Nonetheless, the natural differences in population sizes as previously observed in the valley [12].

While the work with community volunteers certainly increased the ability of research team to identify the *An. funestus* swarms, visual estimates of the swarm sizes by the volunteers did not strongly correlate with the sweep net estimates (R = 0.4518). Though the volunteers were able to locate *An. funestus* swarms, either they could not accurately estimate the number of mosquitoes in the swarm, or the flying males were able to avoid the sweep nets. In the previous study however, there was a strong correlation between sweep nets estimates and visual estimates of the number of *An. arabiensis* in the swarm, indicating that visual estimates could possibly be relied upon to estimate swarm sizes [34]. Given that this current study was not done across multiple seasons, the discordance between estimates may indicate density dependence of such relationships, or that the correlations are non-linear (Fig. 3). Besides, one limitation of the approach is that the approximation of the peak swarming time and the collections using sweep nets may have been imprecise, even though the standardization allowed comparison across all the swarming events.

The molecular analysis confirmed that 100% of all amplified samples of *An. funestus* s.l. mosquitoes were *An. funestus* sensu stricto (s.s.). Although *Anopheles rivulorum* and *Anopheles leesoni* have also been recorded from this area [12], the present study did not find any other sibling species in the swarms apart from *An. funestus* s.s. However, in a recent study in Zambia, a mixed swarm of *An. funestus* and *An. leesoni* was found [20]. Though rare, *An. arabiensis* and *An. funestus* mosquitoes may also swarm either in very close proximity or together. Indeed, in the previous study of *An. arabiensis* swarms, one instance of 13 male *An. funestus* mosquitoes was observed in a sweep net targeting the former species [34]. Also, in this current work, four instances of 2, 2, 3 and 7 *An. arabiensis* males were caught in a sweep net targeting *An. funestus*. Previously, mixed swarms of *Anopheles coluzzii*, *An. gambiae* and *An. funestus* have also been reported from other areas in Africa [20, 42].

Genitalia rotation is a physiological change that occurs when male mosquitoes become sexually mature [20]. Dahan and Koekemoer indicated that these are visible a few hours after emergence, but the rotation rate can increase with the increase in temperature [38]. During this process, the genitalia turn clockwise or ant-clockwise until sexual maturity is reached at 180 degrees full rotation. This current study showed that 100% of all sampled males had complete genitalia rotation, suggesting that only sexually mature males participate in the swarming activity.

It was also observed that there was no special selection based on size of mosquitoes entering the swarms. The wing lengths of *An. funestus* males ranged between 2.0 and 2.8 mm, with no statistically significant differences observed in mean sizes between the mosquitoes caught in the swarms and those caught resting indoors. The results are comparable with those of Charlwood et al. [31] on *An. funestus* in Mozambique.

The elusive nature of *Anopheles* swarms in East Africa is confirmed the paucity or complete lack of reports on such swarms by previous entomologists working in the region. However, this study adopted the approach of working with trained community members to search for swarms as previously described by Kaindoa et al. [34], and also in Burkina Faso [27]. Similar approaches have also been used by community members to accurately identify places with low, medium and high mosquito densities [43]. This has implications for vector control strategies using community participation in targeting mosquito swarms. Indeed, there are several examples where community participation in vector control programs have been successfully relied upon for disease control [44–46]. Even though aerosol spraying could be used to target swarms, additional surveys are needed given that *An. funestus* swarms occur very close to human houses and are generally smaller than *An. arabiensis* swarms. Moreover, additional safety precautions would be required to protect humans. Alternatively, improved technologies such as small robotic drones could potentially be used to target the identified swarms of *An. funestus* mosquitoes, and apply small but targeted insecticide doses.

The challenge of identifying *An. funestus* swarms in the study area was associated with the unexpected low height of the swarms, given that this study had used previous knowledge of *An. arabiensis* swarms when searching for *An. funestus* swarms. *Anopheles funestus* swarms occurred in close proximity to the houses, places that could not be predicted or associated with mosquito swarming. Additional research and exploration of technologies such as the use of unmanned aerial vehicles fitted with high-resolution infrared cameras could help to locate swarms in areas that are inaccessible by humans. Moreover, though the characterization here was restricted to one season, future studies should consider assessments across multiple seasons to assess whether climatic factors may have an influence on the characteristics of these *An. funestus* swarms. There is also a need to develop methods for prediction and estimation of *An. funestus* swarms, which could help to improve the control of malaria in rural areas.

## Conclusion

This study has demonstrated for the first time the occurrence of *An. funestus* swarms in south-eastern Tanzania. Based on available evidence, the study team believe that this is also the first report on swarms of *An. funestus* in the country. More intensive studies should be conducted so as to map and characterize *An. funestus* swarms and assess the factors which influence swarming and mating behaviours of this species as well as how these swarms could best be targeted for control. Approaches aimed at *An. funestus* swarms could be one of the complementary tools used alongside existing interventions, such as long-lasting insecticide-treated nets (LLINs) and indoor residual spraying (IRS) to control malaria transmission, to target the mosquitoes while outdoors.

## Abbreviations

ACT: artemisinin-based combination therapy
GLM: generalized linear model
IRS: indoor residual spraying
LLINs: long-lasting insecticide-treated nets
PCR: polymerase chain reaction
